# Reexamining Health-Care Coalitions in Light of COVID-19

**DOI:** 10.1017/dmp.2020.431

**Published:** 2020-11-04

**Authors:** Daniel J. Barnett, Lauren Knieser, Nicole A. Errett, Andrew J. Rosenblum, Meena Seshamani, Thomas D. Kirsch

**Affiliations:** 1 Department of Environmental Health & Engineering, Johns Hopkins Bloomberg School of Public Health, Baltimore, Maryland, USA; 2 Audacious Inquiry, Baltimore, Maryland, USA; 3 Department of Environmental and Occupational Health Sciences, University of Washington School of Public Health, Seattle, Washington, USA; 4 The Johns Hopkins University, Baltimore, Maryland, USA; 5 Clinical Care Transformation, MedStar Health, Washington, DC, USA; 6 The National Center for Disaster Medicine and Public Health and the Department of Military & Emergency Medicine, Uniformed Services University of the Health Sciences, Bethesda, Maryland, USA

**Keywords:** health-care coalitions, COVID-19, hospital preparedness program

## Abstract

The national response to the coronavirus disease 2019 (COVID-19) pandemic has highlighted critical weaknesses in domestic health care and public health emergency preparedness, despite nearly 2 decades of federal funding for multiple programs designed to encourage cross-cutting collaboration in emergency response. Health-care coalitions (HCCs), which are funded through the Hospital Preparedness Program, were first piloted in 2007 and have been continuously funded nationwide since 2012 to support broad collaborations across public health, emergency management, emergency medical services, and the emergency response arms of the health-care system within a geographical area. This commentary provides a SWOT (strengths, weaknesses, opportunities, and threats) analysis to summarize the strengths, weaknesses, opportunities, and threats related to the current HCC model against the backdrop of COVID-19. We close with concrete recommendations for better leveraging the HCC model for improved health-care system readiness. These include better evaluating the role of HCCs and their members (including the responsibility of the HCC to better communicate and align with other sectors), reconsidering the existing framework for HCC administration, increasing incentives for meaningful community participation in HCC preparedness, and supporting next-generation development of health-care preparedness systems for future pandemics.

Despite almost 2 decades of federal investment, the coronavirus disease 2019 (COVID-19) pandemic has again demonstrated the limitations of health-care and public health preparedness in the United States. Health-care and public health system readiness attracted increased national attention after the 2001 9/11 terrorist attacks when the Federal government began funding programs, such as the Strategic National Stockpile (SNS), the National Bioterrorism Hospital Preparedness Program (NBHPP), the Public Health Emergency Preparedness Program (PHEP), and the Metropolitan Medical Response Program (MMRS). The Centers for Disease Control and Prevention’s (CDC’s) PHEP cooperative agreement provides funds to state, territorial, and local health departments to improve preparedness for public health emergencies. The NBHPP ultimately became the Hospital Preparedness Program (HPP), now administered by the Office of the Assistant Secretary for Preparedness and Response (ASPR), and remains the only continuously appropriated source of federal funding for health-care system readiness.^1^ Unfortunately, the budgets of both the HPP and PHEP have been reduced by half (in real dollars) over the past 2 decades.^2^

A central aim of the HPP is to establish health-care coalitions (HCCs), which are partnerships among public health agencies, hospitals, emergency medical services (EMS), emergency management agencies, and other entities to prepare for and respond to emergencies in their jurisdiction.^3,4^ HCCs are intended to “*coordinate activities … to ensure each member has what it needs to respond to emergencies and planned events, including medical equipment and supplies, real-time information, communication systems, and educated and trained healthcare personnel*.” While several HCCs have made a significant impact on local and state response over the years,^5^ the efficacy of HCCs on a national scale remains uncertain. The national response to COVID-19 has illuminated critical gaps in hospital surge management,^6^ insufficient supply and inequitable distribution of personal protective equipment (PPE) and other critical supplies,^7^ delayed infection control procedures,^8^ lack of operational collaboration across the health-care community,^9^ and outdated information and data sharing platforms,^10^ all roles that HCCs were designed to take on.

While it is clear that hospitals and health-care systems are under-equipped to face complex infectious disease threats, it is less clear how HCCs may be better supported and better leveraged to address these challenges. This commentary uses a “SWOT analysis” framework (Table 1) to summarize the internal *strengths* and *weaknesses*, as well as external *opportunities* and *threats* related to the HCC model. It closes with concrete recommendations for improving health care system readiness and increasing response capacity through the HCC model.

**Table 1.** Strengths, weaknesses, opportunities, and threats for HCCs in light of COVID-19

Strengths	Weaknesses
HCCs build relationships to strengthen response to emergencies that impact the health-care system, including through: Enhanced interoperability among organizations Open sharing of resources and information Improved communication among agencies and the public. HCCs may have more political influence than their individual member organizations. HCCs provide a forum for sharing knowledge, skills, and abilities, which can reduce duplication of effort, increase standardization, and achieve more significant economies of scale. HCCs can facilitate vertical and horizontal intergovernmental coordination among core members (hospitals, public health agencies, EMS, and emergency management) and with other components of the health care sector (eg, behavioral health services, postacute care facilities, medical device manufacturers and distributors, etc.).	HPP funds are administered by state and territorial health departments, which have little influence on the delivery of care or the allocation of health-care resources during a disaster and cannot compel health-care organizations to participate in HCCs. Many HCCs are coordinated by chronically underfunded health departments with insufficient resources to lead health-care response in addition to more traditional responsibilities in a disaster. HCCs and their members often have differing response missions and capabilities and the degree of meaningful participation in HCCs remains unclear. HCCs seeking to work across geopolitical boundaries may be limited by being situated within state and local health departments.
Opportunities	Threats
Public and political attention on gaps in health-care system readiness may: Create momentum behind ASPR’s plans to create “a ‘national disaster health care system’” announced prepandemic.^15^ Lead to more sustainable funding mechanisms not solely reliant on federal sources, for example, linking HCC participation to accreditation/reimbursement requirements. Result in better alignment across HPP, PHEP, and Homeland Security Grant Program requirements. Encourage HCCs to recognize, exercise, and improve their operational response roles and to coordinate and distribute health-care resources more proactively.	HCCs’ reliance on annual and variable congressional appropriations prevents long-term preparedness planning and infrastructure investments. Event-specific infusions of federal funds (eg, following Hurricane Sandy or the 2014-16 Ebola outbreak) have not been sustained, resulting in the steady loss of many of the achieved gains.

ASPR, Assistant Secretary for Preparedness and Response; COVID-19, coronavirus disease 2019; EMS, emergency medical services; HCC, health-care coalition; HPP, Hospital Preparedness Program; PHEP, Public Health Emergency Preparedness Program.

## Strengths of HCCs

The greatest strength of HCCs is summarized in their name—they are a coalition of disparate public and private organizations whose collective mission is to minimize disruption of health-care delivery during disasters and public health emergencies. Today, there are over 31,000 HCC members nationwide, and 85% of hospitals, 82% of local health departments, 56% of emergency management organizations, and 27% of EMS agencies participate in a HCC.^3^ Research among high-performing HCCs has found that their greatest value is the community and regional partnership that enables interoperability among organizations, open sharing of resources and information, and improved communication among agencies and the public.^11^ As an additional benefit, these cross-agency partnerships may be more competitive for funding opportunities and to exert political influence than any 1 agency would be alone.

HCCs also provide a forum for shared knowledge, skills, and abilities for the participating organizations, which can reduce duplication of effort, increase standardization, and achieve more significant economies of scale. This could include pooling expertise in areas such as trauma, pediatrics, burn, and mental health. For example, a HCC might designate 1 participating agency as the regional HAZMAT lead to provide training and resources for frontline responders across multiple organizations, rather than each organization developing this capacity separately. This could also include the pooling of specialized systems and technologies, for example, a centralized system for health-care provider credentialing and privileging across facilities or a shared telehealth infrastructure.

Another strength of HCCs is the ability to better coordinate and communicate across regions and at the state and federal levels. During an emergency, such as a pandemic, the regional alignment of staff, stuff, space, and systems can be critical to a successful response. Identification of a unified point of contact for state and federal resources can streamline communication.

## Weaknesses of HCCs

The HPP cooperative agreements are administered by state and territorial health departments, not by the hospitals and health systems that will provide care during a disaster. While this arrangement facilitates the distribution of federal funds across state and territorial governments, it also has several disadvantages. First, public health departments have little control or influence on the delivery of care or allocation of health-care resources during a response. Health departments cannot compel a health-care organization to meaningfully participate in a HCC, and while HPP reports that 85% of hospitals are HCC members,^3^ the level and degree of meaningful participation is unclear.^12^ Decreasing federal funding through HPP and PHEP means that public health departments also lack access to the financial capital that may be required to more effectively incentivize health-care organizations to meaningfully participate in preparedness activities. Second, state and local public health agencies are chronically underfunded,^2^ and have decreasing staff to meet increasing demands for even their core public health services.^13^ Adding health-care readiness to their traditional roles can overburden employees whose primary responsibilities are to their full-time position and not those of the HCC as a collective.^11^ Finally, health-care delivery systems often do not align with the geopolitical boundaries served by public health agencies because they cross city, county, and even state borders.^14^ Funding state and territorial public health agencies rather than directly funding health-care organizations limits the ability to conduct preparedness and response activities that transcend political boundaries. For example, many of the health-care organizations in the National Capital Region span the District of Columbia and multiple counties in Virginia and Maryland, yet preparedness and response activities are often handled separately and differently by each of these jurisdictions, increasing complexity of planning and execution for these organizations.

## Opportunities for HCCs

The COVID-19 response has brought national attention to the need for greater investment in health-care system readiness. Given the collective acknowledgement of existing deficiencies, there is now a critical opportunity to create more sustainable funding mechanisms for HCCs. Even before the pandemic, ASPR announced plans to create “a ‘national disaster health care system’ by better leveraging and enhancing existing, such as the Hospital Preparedness Program (HPP) and the National Disaster Medical System (NDMS, to create a more coherent, comprehensive, and capable regional system integrated into daily care delivery.”^15^ Recognizing that a sustainable funding model cannot rely solely on waning federal sources, ASPR also investigated potential incentives for meaningful and sustainable health-care sector investment in readiness,^16^ for example, by linking coalition participation to hospital accreditation and/or reimbursement requirements. However, to date, these potential incentives have yet to be implemented.

The acute strain on health-care facilities in jurisdictions that are grappling with a surge of COVID-19 patients has also drawn attention to the need for a more robust national health-care response capability. Although the 2017-2022 Health Care Preparedness and Response Capabilities (HCPR Capabilities), published by ASPR as a guide for the health-care delivery system (including HCCs), includes several capabilities and objectives related to direct provision of health care and surge management,^17^ national capability remains suboptimal. While some HCCs have developed significant operational response capabilities,^18^ many others see themselves as a resource support network^19^ or planning entity^20^ rather than an active partner in response. The COVID-19 response has provided a critical opportunity for HCCs to recognize, exercise, and improve their operational response roles and to coordinate and distribute health-care resources more proactively.

The COVID-19 response has also graphically illustrated the intersection of clinical health, public health, and socioeconomic dependencies and the importance of bringing diverse professional sectors together as HCCs were created to do. The HCPR Capabilities require participation of 4 core HCC members, that is, hospitals, public health agencies, EMS, and emergency management agencies, and suggest a plethora of others from within the health-care sector (eg, behavioral health services, postacute care facilities, medical device manufacturers and distributors, etc.) and outside of it (eg, nongovernmental organizations, public safety, schools/universities, faith-based organizations, etc.). A salient opportunity for greater HCC effectiveness is through better cross-sector collaboration through the meaningful engagement of other sectors interested and invested in disaster risk reduction, as well as with sectors upon which the health-care sector is critically dependent. This may be achieved, in part, through the intentional alignment of requirements across the HPP, PHEP, and Homeland Security Grant Programs,^21^ as well as across other federal health and health-care grant programs that are not traditionally focused on emergency response to promote greater collaboration and additional clarity around roles in response.

## Threats to HCCs

The biggest threat to HCCs is insufficient, precarious, and diminishing funding. The COVID-19 pandemic has highlighted the impacts of chronic under-resourcing of public health and health-care emergency preparedness. In a country that spends approximately $3.6 trillion per year on health, the annual HPP budget has been cut from $515 million in 2003 to $275.5 million this year.^2,22,23^ Proportional declines in appropriations have been experienced by the PHEP cooperative agreements that fund state and local public health preparedness and response programs.^2^ The steady decline of federal funding has hampered health-care preparedness progress and has reduced the financial leverage to convince hospital leadership to participate.

The reliance on annual and variable congressional appropriations prevents long-term preparedness planning and infrastructure investments. Historically, prior disasters have led to large, transient infusions of federal event-specific funding. This year, the $2 trillion federal Coronavirus Aid, Relief, and Economic Security (CARES) Act provided more than $130 billion to hospitals, health-care systems, and providers.^24^ But these have been 1-time investments, not sustained increases in health preparedness funding. The result is a short-term spike in resilience, followed by the steady loss of many of the achieved gains.

## Recommendations

In the coming years, climate change and urbanization will lead to more frequent and severe disasters. The natural history of influenza and other infectious diseases suggests the likelihood of future pandemics, and the unprecedented speed and impact of the COVID-19 outbreak have demonstrated that our health-care system needs to recalibrate for this new era. At present, HCCs have been the only continuously federally supported program designed to promote coordinated preparedness and response of our largely private and disjointed health-care system. By this fact alone, their sustainability is imperative. We present here 4 recommendations for strengthening HCCs for future pandemics:

### Recommendation 1: Better evaluate the roles and responsibilities of HCCs and their members in disaster response to unify efforts and improve accountability

When functioning to their fullest, HCCs are a prime example of “the whole is greater than the sum of its parts.” The breadth and saturation of stakeholder engagement in HCCs today indicates their capacity to bring together diverse organizations with a broad array of skills and capabilities. However, the current lack of standardization of HCCs’ structures, forms, and functions may hinder meaningful participation, program-wide evaluation, and accountability. The role of the HCC as a collective and that of each of its discrete members needs to be clearly defined and continuously evaluated as a requirement for continued funding. Attention should be given to creating a truly holistic approach, that includes not only hospitals and health-care partners as members, but also members of other sectors with a vested interest in readiness, with bidirectional communication between members that creates more cohesive preparedness. State and local emergency management and incident command structures should also consider formally integrating HCCs into the existing response infrastructure. For example, HCCs could provide leadership and support in Medical Operations Coordination Cells (MOCCs) within sub-state regional and statewide Emergency Operations Centers.

### Recommendation 2: Reconsider existing frameworks for HCC administration

Administering HCC funds through state and local health departments thwarts private investment in regional health-care system preparedness, increases demands on already emaciated health departments, and requires them to coordinate hospitals and health-care facilities that have little incentive to participate or comply with plans and policy recommendations. The federal government should consider alternative frameworks for HCC administration that may achieve a better balance between public health and health-care leadership, enable HCCs to pursue alternative revenue streams, and improve the health-care system’s investment in and commitment to resilience. There are several options for how this could be done, for example, by establishing within states an independent nonprofit organization to administer HPP funds or directing funds to a health-care organization with a high level of established engagement across the health-care sector (eg, hospital associations).

### Recommendation 3: Create incentives for more meaningful hospital and health system leadership in HCCs

Ultimately, hospitals and health-care systems have a lot to lose in a disaster. In addition to the human toll of a pandemic on frontline health-care workers,^25^ the financial damage of disruptions to nonemergency care can be significant. HCCs stand poised to help mitigate these losses with robust preparedness. Thus, hospitals and health systems should be incentivized to further support HCCs. For example, Medicare reimbursements could be adjusted for health-care systems that contribute to HCCs. Additional sources of funding should also be explored and encouraged, such as providing tax incentives or insurance benefits for increased private sector investments.

### Recommendation 4: Support the next generation of US health-care preparedness systems

ASPR’s goal to create a next-generation national disaster health-care system provides an opportunity to rethink health care system readiness priorities. While funding for HCCs supports increased collaboration and communication across health care organizations that are critical to disaster response, it does not adequately focus on improving the underlying systems that support HCCs and the health-care and public health communities. As the COVID-19 response has highlighted, these systems, including telehealth, incident management, surge capacity dashboards, surveillance systems, immunization registries, and contact tracing databases, are woefully inadequate for large-scale response efforts.^6,8-10^ The next generation of US health-care preparedness systems should be built to leverage modern technology and invest as much in functional systems as it does in stakeholder engagement.

The COVID-19 pandemic has again shown the limitations of our public health and health-care system in a disaster. While HCCs provide a foundation for national health-care system response capabilities, their essential role in response has not been adequately resourced or evaluated. Taken collectively, the provided recommendations aim to strengthen the existing HCC model by calling attention to the infrastructure and incentives needed to achieve more widespread buy-in and collaboration of clinical health-care stakeholders in readiness activities. Critical changes in funding, organization, and activities are needed to improve our national health-care resilience and prepare us for the next big public health emergency or disaster.
